# Epidemiological Characteristics and Regional Risk Prediction of Hemorrhagic Fever with Renal Syndrome in Shandong Province, China

**DOI:** 10.3390/ijerph18168495

**Published:** 2021-08-11

**Authors:** Kaili She, Chunyu Li, Chang Qi, Tingxuan Liu, Yan Jia, Yuchen Zhu, Lili Liu, Zhiqiang Wang, Ying Zhang, Xiujun Li

**Affiliations:** 1 Department of Biostatistics, School of Public Health, Cheeloo College of Medicine, Shandong University, Jinan 250012, China; kailishe95@163.com (K.S.); lichunyu_biosta@163.com (C.L.); chanzyq@163.com (C.Q.); 201935820@mail.sdu.edu.cn (T.L.); jiayan8hui@163.com (Y.J.); zhuyuchenl@163.com (Y.Z.); lili160629@163.com (L.L.); 2 Institute of Infectious Disease Control and Prevention, Shandong Center for Disease Control and Prevention, Jinan 250014, China; wzq3678@126.com; 3 Faculty of Medicine and Health, School of Public Health, University of Sydney, Camperdown, NSW 2006, Australia; ying.zhang@sydney.edu.au

**Keywords:** hemorrhagic fever with renal syndrome, epidemiological characteristics, regional risk prediction, boosted regression tree model, influencing factors

## Abstract

Background: Hemorrhagic fever with renal syndrome (HFRS), a rodent-borne disease caused by different species of hantaviruses, is widely endemic in China. Shandong Province is one of the most affected areas. This study aims to analyze the epidemiological characteristics of HFRS, and to predict the regional risk in Shandong Province. Methods: Descriptive statistics were used to elucidate the epidemiological characteristics of HFRS cases in Shandong Province from 2010 to 2018. Based on environmental and socioeconomic data, the boosted regression tree (BRT) model was applied to identify important influencing factors, as well as predict the infection risk zones of HFRS. Results: A total of 11,432 HFRS cases were reported from 2010 to 2018 in Shandong, with groups aged 31–70 years (81.04%), and farmers (84.44%) being the majority. Most cases were from central and southeast Shandong. There were two incidence peak periods in April to June and October to December, respectively. According to the BRT model, we found that population density (a relative contribution of 15.90%), elevation (12.02%), grassland (11.06%), cultivated land (9.98%), rural settlement (9.25%), woodland (8.71%), and water body (8.63%) were relatively important influencing factors for HFRS epidemics, and the predicted high infection risk areas were concentrated in central and eastern areas of Shandong Province. The BRT model provided an overall prediction accuracy, with an area under the receiver operating characteristic curve of 0.91 (range: 0.83–0.95). Conclusions: HFRS in Shandong Province has shown seasonal and spatial clustering characteristics. Middle-aged and elderly farmers are a high-risk population. The BRT model has satisfactory predictive capability in stratifying the regional risk of HFRS at a county level in Shandong Province, which could serve as an important tool for risk assessment of HFRS to deploy prevention and control measures.

## 1. Introduction

Hemorrhagic fever with renal syndrome (HFRS), a rodent-borne zoonotic disease caused by hantaviruses, is transmitted to humans mainly via an aerosolized virus that is shed in urine, feces, and saliva [1]. Infected individuals often develop clinical symptoms characterized by fever, circulatory collapse with hypotension, hemorrhage, and acute kidney injury [2]. To date, there is no specific therapy for HFRS. Supportive therapy is the mainstay for patients [3]. Long-term outcomes of HFRS have been reported as a higher risk of hypertension, acute myocardial infarction, stroke, venous thromboembolism, lymphoma, and so on [4,5,6,7]. In addition, kidney dysfunction caused by HFRS can also cause cardiorenal syndrome, which may increase readmission rate, intensive care, and costs [8,9].

Hantaviruses are distributed worldwide and pose a serious threat to public health. It is estimated that there are more than 150,000 cases of HFRS worldwide each year, with most occurring in Eurasia [10]. China is the most endemic country, and the main pathogens of HFRS are Hantaan virus (HTNV) and Seoul virus (SEOV). From 2004 to 2019, there were 209,209 HFRS cases and 1855 deaths reported in mainland China, resulting in a significant disease burden [11]. After the implementation of comprehensive control activities, such as disease surveillance, rodent eradication, and vaccination immunization, the incidence and fatality rate of HFRS in China have declined significantly. However, the situation of HFRS is still serious, and an upward trend has been observed in several regions recently [12]. Shandong, a province with a large population of more than 100 million, is one of the most seriously affected areas in China. In recent years, the number of cases in Shandong ranked the top five among all provinces in China, raising public health concerns [12,13].

The epidemic of HFRS is the consequence of the interaction between virus, reservoir hosts, and individual humans [14], which could be affected by meteorological, geographical environment, socioeconomic status, healthcare systems, and other factors. Previous studies have demonstrated that temperature, precipitation, and relative humidity have significant effects on the abundance and distribution of host animals. Therefore, all these factors may influence the transmission of hantaviruses [15,16]. The normalized difference vegetation index (NDVI), land use, and topography have been reported as closely associated with the incidence of HFRS [17,18,19]. Moreover, owing to the advancing economy, together with the expansion of human activities (e.g., population growth and migration, agricultural and forestry activities, urbanization, etc.), hantavirus epidemics are also prolonged in urban regions [20]. However, the influencing factors considered in previous studies are relatively narrow, most of which are mainly meteorological factors, while few studies comprehensively take all the above factors into account. Meanwhile, conventional methods are usually limited by interaction and collinearity that may influence quantifying variable effects.

The boosted regression tree (BRT) model is a machine learning method that combines the advantages of regression tree algorithm and boosting algorithm. It can examine complex linear and nonlinear responses, including interactions between predictors, and is relatively insensitive to collinearity problems [21,22]. BRT has been widely used in the field of infectious diseases [23,24,25]. However, previous studies have mainly focused on the exploration of risk factors for disease with limited efforts on predicting risks at a small scale. Therefore, this study is innovative in applying the BRT model to explore the effects of environmental and socioeconomic factors on HFRS and to forecast HFRS risk zones in Shandong Province.

This study aims to provide a comprehensive analysis of HFRS epidemiological characteristics from 2010 to 2018 in Shandong Province, to identify important influencing factors of HFRS and to map infection risk zones at the county level. Our findings will assist future targeted surveillance and control efforts to prevent HFRS.

## 2. Materials and Methods

### 2.1. Study Area

**Figure 1:**
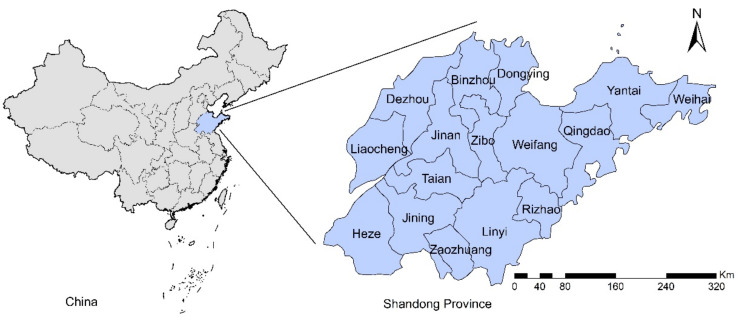
The geographical location of Shandong Province.

Shandong, a coastal province in eastern China, is located between latitude 34°23′ and 38°24′ N and longitude 114°47′ and 122°42′ E (Figure 1). Shandong Province has 16 cities and 137 counties or districts, with a population of 100,702,100 and an area of 157,965 km^2^. It is characterized by a monsoon climate with four distinct seasons. The average annual temperature ranges from 11 °C to 14 °C, and the average annual precipitation is generally between 550 mm and 950 mm.

### 2.2. Data Sources

The epidemic data of HFRS from January 2010 to December 2018 were extracted from the China Information System of the Shandong Center for Disease Control and Prevention. Cases information on age, sex, occupation, symptom onset time, and located county were obtained. All the data were in an anonymous format. According to the “Diagnostic criteria for epidemic hemorrhagic fever (WS278-2008)” issued by the Chinese Ministry of Health, a probable HFRS case is defined as a patient with fever, gastrointestinal symptoms (e.g., nausea, vomiting, abdominal pain, etc.), and manifestations of capillary damage (e.g., hyperemia, exudation, and hemorrhage) who either had a history of living in the epidemic area or had a direct or indirect contact history with the infected rodents or their excreta and secretions within 2 months before onset. A clinically diagnosed case is defined as a probable case with clinical or laboratory evidence meeting one or more of the following criteria: (1) hypotension shock or renal damage, (2) leukocytosis and thrombocytopenia in peripheral blood during febrile phase, (3) a positive urinary protein, (4) elevated serum creatinine and urea nitrogen. A laboratory confirmed case is defined as a probable case or a clinically diagnosed case with laboratory evidence meeting one or more of the following criteria: (1) a positive result for the serum-specific IgM antibody, (2) more than four-fold increment for the serum-specific IgG antibody in convalescent period compared to in acute period, (3) hantavirus RNA detected from the patients, (4) hantavirus isolated from the patients. In this study, we included clinically diagnosed and laboratory confirmed cases. Demographic data for the same period in each county were collected from Shandong Provincial Bureau of Statistics (http://tjj.shandong.gov.cn/, accessed on 4 January 2021). The incidence for each county was calculated by the epidemic reported data and population data.

Based on what has been reported in previous literature, we selected a set of raster data of environmental and socioeconomic variables that might contribute to the incidence of HFRS. Meteorological factors, including annual average temperature and annual cumulative precipitation, were derived from the National Earth System Science Data Center, National Science & Technology Infrastructure of China (http://www.geodata.cn, accessed on 29 December 2020). The elevation of the land (later, “elevation” only), NDVI, land use, and gross domestic product (GDP) data were downloaded from the Resource and Environment Science and Data Center of the Chinese Academy of Sciences (http://www.resdc.cn/, accessed on 30 December 2020). For land use data, percentage coverage of cultivated land, woodland, grassland, water body (e.g., rivers, lakes, etc.), urban land, and rural settlement were summarized at the county level. Detailed information of related environmental and socioeconomic factors are listed in Table S1.

### 2.3. Statistical Analysis

A BRT model was employed to identify important influencing factors of HFRS and to forecast risk zones in our study. This model is effective for accommodating complex response functions while avoiding overfitting. It combines regression or classification trees with boosting algorithms that firstly fit a regression tree and then iteratively improve accuracy in a forward stage-wise fitting process by reducing variance in the response [21,26]. The final BRT model can be viewed as an additive model of many trees.

The BRT model depends on three parameters: bag fraction, learning rate, and tree complexity [21]. The bag fraction specifies the proportion of randomly drawn samples to be used for modeling in each iteration. The learning rate determines the contribution of each new tree to the model. The tree complexity indicates the number of nodes in a tree, which can control the interaction between the predictors. These parameters in the current study were designated at 0.75, 0.005, and 4, respectively [23]. A ten-fold cross-validation method was performed to further optimize the number of trees. The area under the ROC curve (AUC) was used to assess the predictive performance of the model.

We firstly categorized the annual incidences into two groups for reasons of overdispersion. Counties with an incidence greater than the annual incidence of Shandong Province were regarded as 1, while those with an incidence less than the annual incidence of Shandong Province were regarded as 0. We fitted the BRT model nine times, each time using one of the years 2010–2018 as the testing data and the remaining years as the training data [27]. Secondly, relative contribution index and partial dependence plots were generated to better explain the results of BRT model. The relative contribution of each predictor is an assessment of variable importance for predicting HFRS risk. These contributions are scaled to sum to 100, with higher numbers indicating a greater effect on the response. In this study, we defined the most significant risk factors as those whose relative contributions were above average levels [24]. Partial dependence plots were used to visualize the predictor–response relationship after averaging the effects of all other predictors [21]. Finally, we used the BRT model constructed with 2010–2017 data to forecast the probabilities for the HFRS risk of each county in 2018, and created a risk map.

All analyses were accomplished with R (version 3.6.3), and “dismo” and “gbm” packages were used to build BRT models [28,29]. The visualization of maps was conducted using the geographical information system (GIS) technique in ArcGIS 10.5 software (ESRI Inc., Redlands, CA, USA).

## 3. Results

### 3.1. Epidemiological Characteristics of HFRS

**Figure 2:**
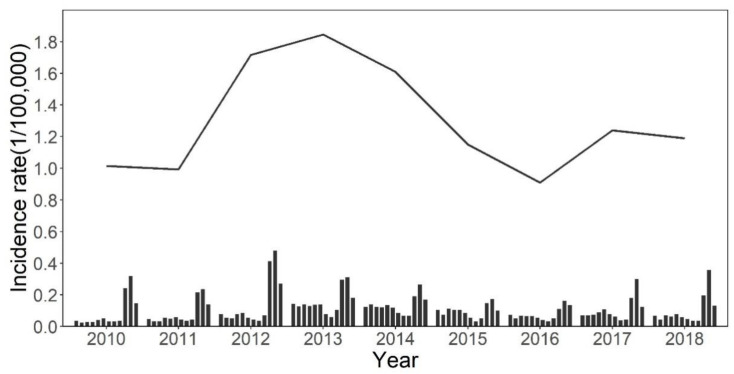
The temporal distribution of HFRS incidence in Shandong Province from 2010 to 2018. Solid lines and bars represent the annual and monthly incidence of HFRS in Shandong Province, respectively.

From 2010 to 2018, a total of 11,432 HFRS cases were reported in Shandong Province, and the annual incidence ranged from 0.91 to 1.84 per 100,000. During the study period, the annual incidence increased from 2010 to 2013, then decreased until 2016, when the incidence climbed up again (Figure 2). The sex-specific annual incidences are shown in Supplementary Figure S1. The annual incidence in males ranged from 1.26 to 2.64 per 100,000, while the annual incidence in females ranged from 0.52 to 1.07 per 100,000. In addition, the monthly incidence showed obvious seasonal characteristics, with two peaks each year; the small peak was from April to June (spring), while the prominent one was from October to December (autumn–winter). The difference between the two peaks was relatively large before 2013, then gradually narrowed from 2013 to 2016, and increased after that (Figure 2).

**Figure 3:**
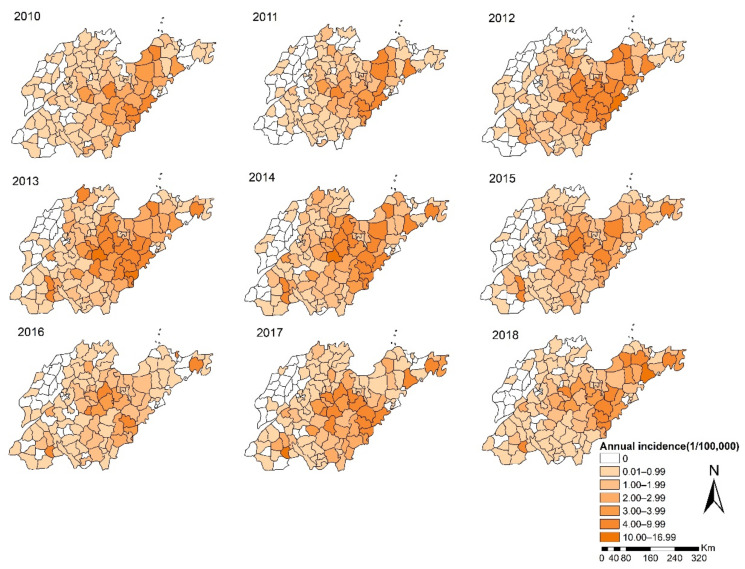
The spatial distribution of HFRS incidence in Shandong Province from 2010 to 2018.

In Figure 3, the spatial distribution of HFRS showed apparent dynamic variation. The high-incidence regions were mainly distributed in the central mountainous and southeastern coastal areas of Shandong Province. During the study period, the process of the expansion and contraction of the high-risk areas was basically consistent with the trend of the annual incidence. From 2010 to 2011, the high-incidence areas were relatively scattered, which converged into a large region during 2012 to 2013, and then separated. It is worth noting that the incidence in some counties of Jining City, located in the southwest of Shandong Province, has remained high since 2012, while surrounding areas are sporadic.

As for demographic characteristics, 72.31% of all cases were males. The median age was 50 years, with the 31–70 years age group (81.04%) being the majority (Table 1). We found that the percentage of the 61–70 years age group increased from 12.96% in 2010 to 19.43% in 2018, while the percentage of the 31–60 years age group decreased (Table S2). The occupations with the highest percentage were farmers (84.44%), followed by workers (5.23%) and students (2.41%) (Table 1).

### 3.2. Influencing Factors for the Epidemic of HFRS

**Figure 4:**
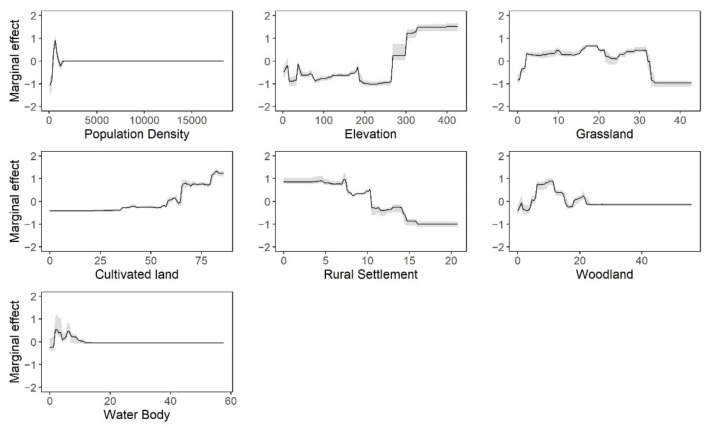
Marginal effect curves of each predictor. The black lines represent the mean effect, and the gray shaded areas represent the range of effect from all BRT models.

Table 2 displays the mean relative contributions of each predictor for HFRS risk, which was derived from nine BRT models. The most significant contributing factors were population density, elevation, grassland, cultivated land, rural settlement, woodland, and water body. As is shown in Figure 4, the relationships between each predictor and the incidence of HFRS were nonlinear. With the increase of population density, the risk of HFRS first increased and then decreased rapidly after reaching the peak. For elevation, the marginal effect fluctuated slightly before 263 m and then increased in step. The effect of grassland was approximately reverse-U-shaped, with the risk of HFRS increasing rapidly before 2.17%, then keeping flat, and decreasing after 31.61%. The cultivated land was positively associated with the risk of HFRS, while rural settlement was negatively related to the risk of HFRS. The effect curves of woodland and water body were similar. Both of them showed an increasing trend first, and then decreasing, followed by a small peak.

### 3.3. Map of HFRS Risk Zones

**Figure 5:**
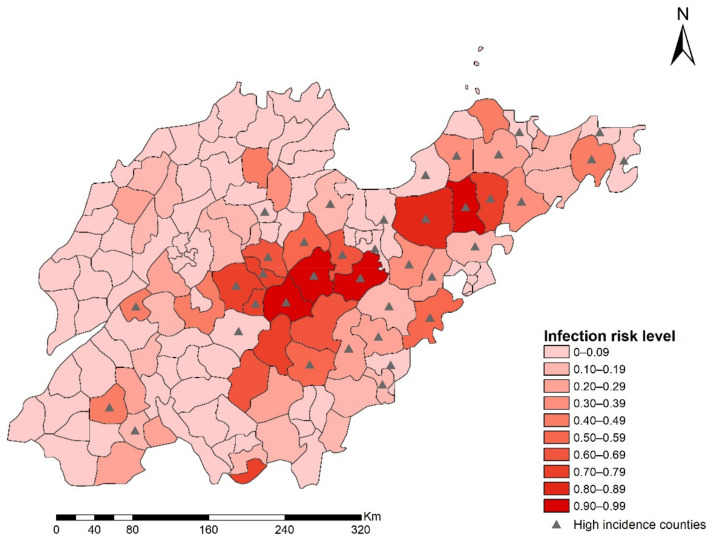
The prediction map of HFRS risk in 2018. The red color represents the probability of being a high-risk county, and the black triangle represents locations of counties whose HFRS incidence exceeded the annual incidence of Shandong Province in 2018.

The mean values of AUCs for training and testing data were 0.910 and 0.912 (Table S3), which indicated that the overall predictive performance of BRT was robust. Based on the estimation from the BRT model, which used 2010–2017 data as the training set, we only plotted a predicted risk map for the year 2018 in Shandong Province (Figure 5). From this map, we can see that counties with an incidence above the provincial-level annual incidence were mainly located in central and eastern areas of Shandong. Meanwhile, the predicted high-risk areas were largely coincident with those mentioned above, which further suggests that the BRT model had an excellent predicting power.

## 4. Discussion

In this study, we illustrated the epidemiological features of HFRS cases and influencing factors, and predicted risk zones in Shandong Province. The annual incidence fluctuated with two incidence peaks each year, which is closely related to the seasonal breeding activity of reservoirs [14]. The small peak in spring is generally caused by high contact rates linked to rodents’ indoor foraging activity, while the autumn–winter peak is mainly attributed to agricultural activities during the harvest season [30,31]. Furthermore, the difference between the two peaks could be caused by changes in the proportion of HTNV-typed HFRS and SEOV-typed HFRS [12].

Sex and age differences in the incidence are consistent with the fact that adult males have more exposure opportunities through occupational and leisure activities. Moreover, due to the migration triggered by young people seeking employment chances out of their home county, more and more older people are involved in agricultural work at home, which increased their risk of infection. These findings are consistent with our previous work [32], which indicated that rural areas are still the focus of prevention and control in Shandong Province. Rodent control, vaccination, and health education should be continually strengthened for middle-aged and older farmers.

We found that there were several high-incidence clusters in Shandong Province, including the central and southeastern part of Shandong, as well some counties in southwestern regions. The coverage of former regions is larger than the latter, and the scope dynamically changed during the study period, suggesting that the focus of surveillance should be timely adjusted according to spatiotemporal dynamic evolution features of epidemic foci.

According to the BRT model, population density, elevation, grassland, cultivated land, rural settlement, woodland, and water body were important influencing factors of HFRS in Shandong Province. The association between population density and HFRS was positive at low levels, whereas a negative correlation was found at high levels. This result is consistent with the results of a study in Shaanxi Province [33], and can be explained as follows. Compared to urban areas, the population density in rural villages is relatively lower. The growth of population in these areas means that the number of vulnerable people increases, and the mismatch in health resources may increase the chance of infection. Second, due to the intensification of human activities, habitat loss led rodents into human settlement, increasing human contact with rodents. However, when the population density reached an urban level, the consequent increase in GDP and improvements in living conditions would protect humans from rodents [34]. Therefore, prevention of HFRS should be further strengthened in rural areas, especially those with relatively high population density.

Although a previous study in China reported that there was a negative relationship between elevation and HFRS [18], this study developed models at the provincial level, and this may impact the accuracy of the results. Given the changing climate and topography within a province, it is necessary to carry out a local study with a more effective model to explore influencing factors for the prevalence of HFRS. We found that the elevation was positively associated with the risk of HFRS, which is consistent with a study in Liaoning Province [35]. In Shandong, the central and southern areas are mainly mountains and hills, and the hantavirus carriage rate of rodents is higher [32]. This may be because the human disturbance is usually limited at higher elevation, which contributes to the growth of vegetation, thereby providing more food for rodents. Additionally, previous research has proved that the ex vivo stability of hantavirus is stronger in lower temperatures [36]. Considering the negative correlation between elevation and temperature, this conclusion can partly explain our results. This further explains why temperature itself is an insignificant factor for the prevalence of HFRS in our model.

Land use could also contribute to the risk of HFRS. Among these variables, medium percentage coverage of grassland and woodland provide convenience for feeding and nesting activities of rodents, making them major sites for rodent population outbreaks. For example, in the study of Xiao [19], the main rodent species in grassland were *R. norvegicus* and *R. flavipectus*, which were the dominant hosts for SEOV. Another interpretation is that grassland and woodland could influence the climate–HFRS association due to ecosystem stability [37]. Even in low rainfall seasons, woodlands could enhance water conservation to safeguard the population of rodents. In addition, cultivated land is another important factor affecting the risk of HFRS. We found that the risk of HFRS increases with increasing coverage of cultivated land, which is consistent with previous work [38]. Cultivated land can provide plenty of food and shelter for rodents. Besides that, long-term field work increased chances for transmission of hantaviruses from rodents to humans. This is why farmers account for the highest proportion of HFRS cases. From the above points, the negative trend for the effect curves of rural settlement can be explained, which owes to the fact that an increase in rural settlement inevitably reduces the coverage of cultivated land and woodland. Regarding the water body, a previous study has qualitatively recognized it as a cause of clusters of HFRS cases [39], and our study provided quantitative evidence. The trend in the fitted curve suggests that a modest increase of water coverage may provide sufficient freshwater resources and a humid living environment for rats. However, a large area of water body will lead to the reduction of habitat, which is not conducive to the survival and reproduction of rodents [40].

For the reason that the transmission of HFRS involves many diverse influencing factors, a statistical method with a high predictive accuracy is desirable. The BRT model is such a model that can not only explore influencing factors but also take advantage of boosting algorithms to obtain robust results [41]. Our results show that the BRT model has satisfactory predictive capability in stratifying the regional risk of HFRS at a county scale in Shandong Province, which provides useful information for the development of preventive measures. The risk map suggests that central and eastern regions of Shandong will remain key epidemic areas. In these areas, public health departments should closely monitor the situation of the epidemic, strengthen the detection of etiology, and allocate medical resources rationally.

This study identified important influencing factors of HFRS and quantified the effects of these factors on HFRS. These results suggest that the implementation of control measures should be tailored to local conditions. For government departments, surveillance of cases and hosts in key endemic districts should be enhanced; rodent prevention and control activities before the two high-risk seasons could be effective in reducing HFRS; health education among key populations such as farmers and foresters should be strengthened, for example, reducing skin exposure and avoiding sitting in the field when farming; the scope of vaccine application in rural villages with high population density should be expanded; there should be a focus on pathogen spectrum and strengthening field investigation in areas with ecological suitability of hantaviruses, particularly in those with relatively high elevation and high vegetation coverage. For individuals, great emphasis should be placed on the use of protective appliances; attention should be paid to the rivers or ponds around living and working places, and self-protection awareness should be increased. In addition, our study detected several high-risk zones with robust estimates, which is useful for disease-related decision-making in different risk zones. For those high-risk areas, corresponding prevention and control measures still need to be strengthened to prevent human infection.

Several limitations existed in this study. First, HFRS cases data were acquired from a passive surveillance system. Thus, underreporting bias was unavoidable. People with mild clinical symptoms may not seek professional medical treatment. Second, our study was conducted at the population level; ecological fallacy limited the capacity for the inference of relationships between the influencing factors and HFRS.

## 5. Conclusions

The epidemic of HFRS in Shandong Province has seasonal and spatial clustering characteristics. The middle-aged and elderly farmers are more susceptible to the infection. Therefore, prevention and control measures should be strengthened for the most vulnerable populations across the province. Population density, elevation, and grassland contributed the most to the epidemic of HFRS in Shandong Province. The predicted high-risk areas were mainly located in central and eastern regions of Shandong, which should be targeted for prioritizing allocation of health resources. The BRT model has satisfactory predictive capability in stratifying the regional risk of HFRS at a county scale in Shandong Province, which could serve as an important tool for risk assessment of HFRS to deploy prevention and control measures.

## Figures and Tables

**Table 1:** Demographic characteristic of HFRS cases in Shandong Province from 2010 to 2018.

Characteristic	Number of Cases	Percentage (%)
Gender		
Male	8267	72.31
Female	3165	27.69
Age (year)		
≤10	62	0.54
11–20	352	3.08
21–30	974	8.52
31–40	1579	13.81
41–50	3048	26.66
51–60	2846	24.90
61–70	1791	15.67
71–80	631	5.52
>80	149	1.30
Occupation		
Farmers	9653	84.44
Workers	598	5.23
Students	275	2.41
Housework and unemployment	222	1.94
Retirees	171	1.50
Others	513	4.49

**Table 2:** Summary of relative contributions of each predictor for HFRS epidemic.

Predictors	Relative Contributions (%)	
	Mean	Range
Population density	15.90	(15.19, 16.50)
Elevation	12.02	(11.50, 13.28)
Percentage coverage of grassland	11.06	(9.99, 11.84)
Percentage coverage of cultivated land	9.98	(9.50, 10.61)
Percentage coverage of rural settlement	9.25	(7.86, 11.67)
Percentage coverage of woodland	8.71	(7.61, 9.54)
Percentage coverage of water body	8.63	(7.35, 11.04)
Annual average temperature	6.20	(4.56, 7.97)
Annual cumulative precipitation	5.42	(4.98, 6.13)
GDP	4.95	(4.18, 6.05)
NDVI	4.57	(3.84, 5.79)
Percentage coverage of urban land	3.31	(2.99, 3.63)

## Data Availability

The data of this study are available from the Shandong Center for Disease Control and Prevention; however, restrictions apply to the availability of the data, which was used under license for the current study and thus cannot be shared publicly.

## Supplementary Materials

The following are available online at https://www.mdpi.com/article/10.3390/ijerph18168495/s1, Figure S1: The sex-specific incidence time series, Table S1: Potential influencing factors considered in the BRT model, Table S2: Distribution of HFRS cases by gender, age, occupation in Shandong Province for each year 2010–2018, Table S3: AUC values for training data and testing data of BRT models.
