# Supplementary material for: Epidemiological Characteristics and Regional Risk Prediction of Hemorrhagic Fever with Renal Syndrome in Shandong Province, China
Source: Int J Environ Res Public Health. 2021 Aug 11;18(16):8495. doi: 10.3390/ijerph18168495 (PMC8391715; doi:10.3390/ijerph18168495)
Supplement: Supplementary file 1 [file ijerph-18-08495-s001.zip › ijerph-1310947-supplementary.pdf]

Epidemiological characteristics and regional risk prediction of hemorrhagic fever with renal syndrome in Shandong Province, China

Kaili She, Chunyu Li, Chang Qi, Tingxuan Liu, Yan Jia, Yuchen Zhu, Lili Liu, Zhiqiang Wang, Ying Zhang and Xiujun Li

Supplementary Material

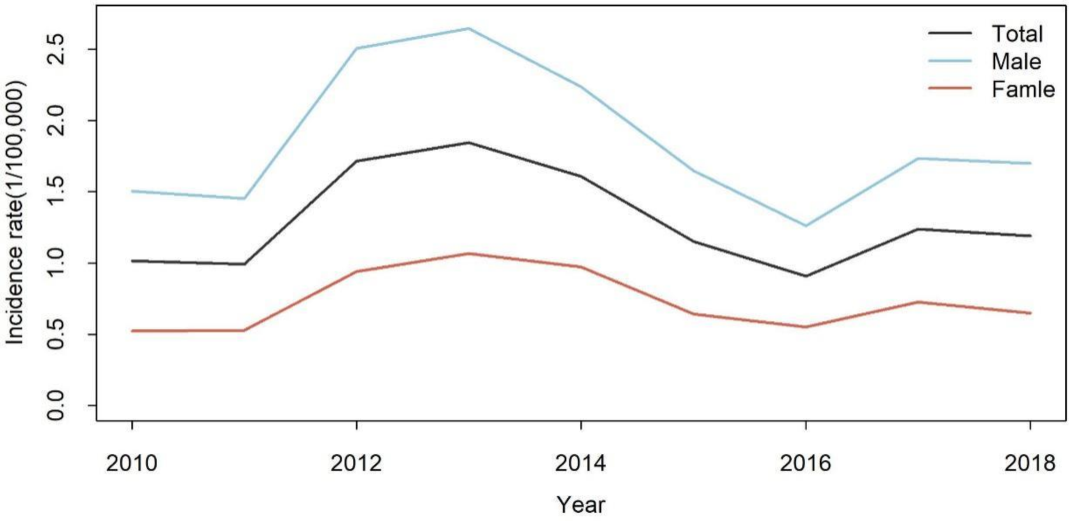

Figure S1. The sex-specific incidence time series.

Table S1. Potential influencing factors considered in the BRT model.

Variables	Description (Unit)	Type
Population density	Population density for each county (person/km^2)	Continuous
Annual average temperature	Annual average temperature for each county (℃)	Continuous
Annual cumulative precipitation	Annual cumulative precipitation for each county (mm)	Continuous
Elevation	Average elevation for each county (m)	Continuous
NDVI	Average normalized difference vegetation index for each county	Continuous
Percentage coverage of cultivated land	Percentage coverage of cultivated land for each county (%)	Continuous
Percentage coverage of woodland	Percentage coverage of woodland for each county (%)	Continuous
Percentage coverage of grassland	Percentage coverage of grassland for each county (%)	Continuous
Percentage coverage of water body	Percentage coverage of water body for each county (%)	Continuous
Percentage coverage of urban land	Percentage coverage of urban land for each county (%)	Continuous
Percentage coverage of rural settlement	Percentage coverage of rural settlement for each county (%)	Continuous
GDP	Average gross domestic product for each county (10,000 yuan/ km^2)	Continuous

Table S2. Distribution of HFRS cases by gender, age, occupation in Shandong Province for each year 2010-2018.

Characteristic	2010	2011	2012	2013	2014	2015	2016	2017	2018	Total
Total, N	972	957	1662	1795	1575	1133	904	1240	1194	11432
Gender, N (%)										
Male	727(74.79)	707(73.88)	1219(73.35)	1291(71.92)	1109(70.41)	823(72.64)	636(70.35)	883(71.21)	872(73.03)	8267(72.31)
Female	245 (25.21)	250(26.12)	443(26.65)	504(28.08)	466(29.59)	310(27.36)	268(29.65)	357(28.79)	322(26.97)	3165(27.69)
Age (year), N (%)										
≤10	6(0.62)	10(1.04)	13(0.78)	7(0.39)	10(0.63)	0(0)	6(0.66)	7(0.56)	3(0.25)	62(0.54)
11-20	33(3.40)	39(4.08)	51(3.07)	48(2.67)	57(3.62)	39(3.44)	24(2.65)	36(2.90)	25(2.09)	352(3.08)
21-30	81(8.33)	80(8.36)	156(9.39)	156(8.69)	143(9.08)	101(8.91)	85(9.40)	86(6.94)	86(7.20)	974(8.52)
31-40	148(15.23)	141(14.73)	268(16.13)	247(13.76)	240(15.24)	140(12.36)	124(13.72)	128(10.32)	143(11.98)	1579(13.81)
41-50	273(28.09)	273(28.53)	451(27.14)	514(28.64)	437(27.75)	313(27.63)	214(23.67)	313(25.24)	260(21.78)	3048(26.66)
51-60	244(25.10)	224(23.41)	396(23.83)	457(25.46)	336(21.33)	264(23.30)	238(26.33)	344(27.74)	343(28.73)	2846(24.90)
61-70	126(12.96)	129(13.48)	246(14.80)	252(14.04)	245(15.56)	197(17.39)	142(15.71)	222(17.90)	232(19.43)	1791(15.67)
71-80	54(5.56)	53(5.54)	70(4.21)	86(4.79)	91(5.78)	64(5.74)	53(5.86)	81(6.53)	78(6.53)	631(5.52)
＞80	7(0.72)	8(0.84)	11(0.66)	28(1.56)	16(1.02)	14(1.24)	18(1.99)	23(1.85)	24(2.01)	149(1.30)
Occupation, N (%)										
Farmers	822(84.57)	831(86.83)	1411(84.90)	1538(85.68)	1325(84.13)	942(83.14)	730(80.75)	1060(85.48)	994(83.25)	9653(84.44)
Workers	62(6.38)	38(3.97)	85(5.11)	95(5.29)	92(5.84)	65(5.74)	59(6.53)	40(3.23)	62(5.19)	598(5.23)
Students	21(2.16)	27(2.82)	45(2.71)	48(2.67)	46(2.92)	25(2.21)	19(2.10)	25(2.02)	19(1.59)	275(2.41)
Housework and unemployment	11(1.13)	11(1.15)	31(1.87)	25(1.39)	28(1.78)	30(2.65)	15(1.66)	34(2.74)	37(3.10)	222(1.94)
Retirees	13(1.34)	8(0.84)	23(1.38)	20(1.11)	18(1.14)	22(1.94)	18(1.99)	24(1.94)	25(2.09)	171(1.50)
Others	43(4.42)	42(4.39)	67(4.03)	69(3.84)	66(4.19)	49(4.32)	63(6.97)	57(4.60)	57(4.77)	513(4.49)

Table S3. AUC values for training data and testing data of BRT models.

Year as testing data	AUC	
	Training data	Testing data
2010	0.908	0.948
2011	0.905	0.927
2012	0.910	0.929
2013	0.913	0.931
2014	0.903	0.953
2015	0.911	0.895
2016	0.911	0.872
2017	0.907	0.923
2018	0.921	0.828
Average	0.910	0.912
